# High-Order Interactions Reshape the Carbon Emission Efficiency Network Across Chinese Regions

**DOI:** 10.3390/e28040431

**Published:** 2026-04-12

**Authors:** Ruijin Du, Xiao Ge, Ziyang Kong, Qingze Shi, Muhammad Ahsan, Lixin Tian

**Affiliations:** 1 School of Mathematical Sciences, Jiangsu University, Zhenjiang 212013, China; xiaos_0421@163.com (X.G.);; 2 Research Institute of Carbon Neutralization Development, School of Mathematical Sciences, Jiangsu University, Zhenjiang 212013, China; 3 Jiangsu Province Engineering Research Center of Industrial Carbon System Analysis, School of Mathematical Sciences, Jiangsu University, Zhenjiang 212013, China; 4 Jiangsu Province Engineering Research Center of Spatial Big Data, School of Mathematical Sciences, Nanjing Normal University, Nanjing 210023, China; 5 Key Laboratory for NSLSCS, Ministry of Education, School of Mathematical Sciences, Nanjing Normal University, Nanjing 210023, China

**Keywords:** carbon emission efficiency, CEE correlation network, simplicial complex theory, high-order interactions, synchronization stability

## Abstract

To address the challenge of balancing economic growth with carbon emission reduction, improving regional Carbon Emission Efficiency (CEE) has emerged as a central pathway to achieving the “dual carbon” goals. While most existing studies focus on inter-regional CEE linkages through pairwise interaction networks, such approaches fall short in capturing the high-order mechanisms of multi-regional collaboration. This study integrates the Super-SBM model with a modified gravity model to construct a CEE correlation network across 30 provincial administrative regions in China from 2007 to 2023. To overcome the limitations of traditional pairwise networks, simplicial complex theory is introduced to establish a high-order topological representation framework. Furthermore, by applying the multiorder Laplacian to assess the synchronization stability of the network, a directed second-order degree swap strategy is proposed to optimize its high-order structure. The findings reveal that the CEE correlation network has evolved from a single-pole aggregation pattern toward a multi-center equilibrium. Provinces with high connectivity play a dominant role in both pairwise and triadic synergies, though their collaborative advantages are gradually diffusing to central and western regions. Notably, with only a limited number (approximately five) of second-order degree swaps among key node pairs, the network’s synchronization stability can be substantially improved. When first-order and second-order interaction strengths reach comparable levels (coupling strength $\alpha^{*}\approx 0.5$), the system achieves optimal resistance to external perturbations. This study highlights the pivotal role of high-order collaboration in shaping regional CEE linkages and offers a practical optimization pathway for structurally enhancing CEE through coordinated efforts in pursuit of the “dual carbon” goals.

## 1. Introduction

Amid profound changes in the global energy structure and the rapid advancement of green and low-carbon transformation, achieving the dual carbon targets has become a key strategic priority for China to foster high-quality development and actively engage in global climate governance [1,2,3]. According to data from the International Energy Agency (IEA) in its Global Energy Review 2025 [4], global energy-related $CO_{2}$ emissions increased by 0.8% in 2024, hitting an all-time high of 37.8 billion tons. Against this severe backdrop, balancing effective carbon emission control with sustained economic growth has emerged as a central challenge for regional sustainable development. Carbon Emission Efficiency (CEE), which measures carbon emission intensity per unit of economic output, serves as a critical indicator of regional green transformation. It not only reflects the efficiency of resource allocation and emission reduction potential but also constitutes the cornerstone for evaluating progress toward low-carbon development. Enhancing CEE is therefore regarded as a vital pathway for reconciling economic growth with emission reduction. Consequently, investigating the linkage mechanisms and collaborative evolution of CEE among regions has attracted increasing attention from both academia and policymakers [5,6,7].

Existing research has produced substantial progress in the measurement of CEE and the identification of its driving factors. Stochastic Frontier Analysis (SFA), Data Envelopment Analysis (DEA), and their extensions are widely applied in CEE evaluation. By presupposing a production function and separating random errors, SFA can accurately measure efficiency levels. Some researchers have employed SFA models to estimate CEE at the city, enterprise, and key industry levels [8,9,10]. In contrast, DEA is favored by researchers for its advantages of not requiring a preset functional form and being able to flexibly handle multiple inputs and outputs. Among its extended models, the Super-SBM model has become a mainstream approach for environmental efficiency assessment due to its ability to effectively handle undesirable outputs and rank efficient decision-making units (DMUs) [11,12,13]. Furthermore, method integration is emerging as a new research trend. Multi-stage DEA models [14], the combination of the undesirable SBM model with the Malmquist index [15], and the integration of machine learning approaches with DEA [16] have been widely applied in efficiency measurement and driving factor analysis in recent years. Meanwhile, numerous studies highlight that CEE is shaped by multiple factors including economic development, industrial structure, technological advancement, energy composition, and policy intervention [17,18,19,20,21,22] and exhibits significant spatial heterogeneity [18,20,23,24,25].

To better capture the interaction among regions, recent scholarship has introduced complex network theory to build CEE correlation networks that reveal the spatial interconnections of CEE. Complex network theory has been demonstrated to be a valuable tool for investigating the structural properties and dynamics of real-world systems across a broad range of disciplines, including physics, ecology, and social sciences [26,27,28,29]. The modified gravity model has been employed to quantify interaction intensity, which, combined with social network analysis (SNA), enabled Wei et al. [30], Zhang et al. [31], Cheng et al. [32], and Du et al. [33] to identify core-periphery structures, clustering features, and key control nodes group in the CEE correlation networks. These works provide new perspectives on collaborative efficiency enhancement. However, they typically assume pairwise interactions, reducing interregional CEE relationships to linear binary correlations, which fails to capture nonlinear collaborative processes involving multiple entities.

Recent developments in complex systems science emphasize that many collective phenomena cannot be adequately explained by pairwise connections alone. From neuronal population synchrony [34] to collaborative innovation in research teams [35] and energy transfer within ecological communities [36], many systems depend on interactions among three or more nodes—so-called high-order interactions. For example, Petri G et al. [34] introduced a “homotopy scaffold” in brain functional networks to reveal high-order information flow structures beyond binary links; Patania A et al. [35] modeled scientific collaboration networks as simplicial complex, transcending dyadic relationships to uncover topological structures formed by multi-author cooperation; and Grilli J et al. [36] demonstrated that in competitive ecosystems, high-order species interactions significantly expand the feasible domain of system stability. Unlike traditional networks that only encode edges, high-order interactions capture the indivisible nature of multi-body collaboration where the outcome of a triplet cannot be reduced to the sum of its pairwise relationships [37,38,39,40]. Simplicial complex theory has thus been integrated into network studies, enabling the direct encoding of irreducible collaborative modules while preserving classical topological information. This framework provides greater expressive power for analyzing clustering, information flow, and systemic stability. At the dynamical level, high-order structures reshape system behaviors. Research indicates that high-order coupling alters thresholds and evolutionary pathways of collective phenomena such as synchronization, diffusion, and phase transitions [41,42,43]. For instance, epidemic spread risks are much higher when three individuals share a room compared with the aggregated risk of dyadic contacts [41]; financial crises intensify when multiple institutions collapse simultaneously, surpassing the sum of isolated failures [42]; and in technological innovation, triadic cooperation networks exhibit greater resilience and creativity compared with bilateral alliances [43]. These insights challenge the binary assumption of traditional networks and propel complex network theory into a “high-order era”.

As a cutting-edge branch of complex systems science, high-order interaction theory has demonstrated strong explanatory power in fields such as neuroscience, ecology, and social dynamics. However, its application in energy-environment systems remains relatively limited. Although existing studies have extensively employed pairwise network analysis to explore the CEE correlation features, a critical research gap persists: no attempt has been made to model high-order correlation structures using simplicial complex theory, nor has there been quantitative analysis of synchronization stability in high-order CEE correlation networks or strategies for its optimization. This gap restricts a deeper understanding of the mechanisms underlying regional collaborative emission reduction and efficiency improvement. The process of enhancing CEE often inherently involves indivisible triadic or even higher-order collaborative relationships, such as the synergistic coordination among technology-exporting provinces, pilot implementation regions, and technology-adopting markets in the advancement of green technologies. In this triad, each role is indispensable and the interaction cannot be reduced to a set of pairwise dynamics. By predominantly relying on pairwise frameworks, current network modeling approaches risk overlooking these key synergistic mechanisms, thereby providing an incomplete picture of the structural dynamics within regional CEE systems.

To address the aforementioned research gaps, this study aims to answer the following three core scientific questions:

- How can we move beyond the pairwise interaction paradigm to construct a high-order CEE correlation network that captures multi-provincial synergies?
- How does the high-order topological structure of this network evolve, and can its synchronization stability be enhanced through structural optimization?
- Is there a universal optimal intervention pathway?

To answer these questions, this paper integrates the Super-SBM model, the modified gravity model, and simplicial complex theory to build a high-order correlation network of CEE across 30 Chinese provinces from 2007 to 2023. A synchronization stability analysis framework based on the multiorder Laplacian is proposed, and a directed second-order degree swap strategy is developed to optimize network structure. The aim is to uncover the high-order organizational mechanisms underpinning collaborative CEE systems, identify critical intervention nodes, and provide a theoretically robust and practically feasible approach for enhancing regional CEE cooperation under China’s dual carbon goals.

## 2. Data and Methodology

### 2.1. Data

The study examines 30 provincial-level administrative regions in China from 2007 to 2023, excluding Tibet, Hong Kong, Macao, and Taiwan. Data on fixed-asset investment and GDP were collected from the China Statistical Yearbook, while labor force and energy consumption figures were obtained from the China Labor Statistical Yearbook and the China Energy Statistical Yearbook, respectively. Carbon emissions data were sourced from the CEADs database (https://www.ceads.net/, accessed on 22 February 2026). Inter-provincial distances were calculated using the R language in combination with the geographical coordinates of provincial capitals. The abbreviations for each province are provided in Table A1 of Appendix A.

### 2.2. Construction of CEE Correlation Network and High-Order Topological Characterization

#### 2.2.1. Carbon Emission Efficiency (CEE) Assessment

Carbon Emission Efficiency (CEE) represents the amount of economic output generated per unit of carbon emissions, thereby indicating how efficiently a region utilizes carbon resources in the course of its economic development [44]. To accurately assess the efficiency of resource allocation in achieving both economic growth and carbon reduction objectives, this study employs the Super-SBM model to evaluate CEE. In this framework, 30 provincial administrative regions of China are considered as decision-making units (DMUs), with fixed asset investment, labor force, and energy consumption serving as inputs, while GDP is treated as the desirable output and carbon emissions as the undesirable output. The mathematical formulation of CEE is expressed as follows [45]:

(1) $$\begin{array}{cc} \; & eff_{i}=\min\frac{1+\frac{1}{3}\sum_{k=1}^{3}\frac{S_{i}^{k-}}{X_{i}^{k}}}{1-\frac{1}{1+1}\left(\frac{S_{i}^{e+}}{y_{i}^{e}}+\frac{S_{i}^{u-}}{y_{i}^{u}}\right)},\;i=1,2,\ldots ,30,\\ \; & s.t.\;\left\{\begin{array}{c} \sum_{j=1,j\neq i}^{n}X_{j}^{k}\lambda_{j}-S_{i}^{k-}\leq X_{i}^{k},\;k=1,2,3,\\ \sum_{j=1,j\neq i}^{n}y_{j}^{e}\lambda_{j}+S_{i}^{e+}\geq y_{i}^{e},\\ \sum_{j=1,j\neq i}^{n}y_{j}^{u}\lambda_{j}+S_{i}^{u-}\leq y_{i}^{u},\\ 1-\frac{1}{1+1}\left(\frac{S_{i}^{e+}}{y_{i}^{e}}+\frac{S_{i}^{u-}}{y_{i}^{u}}\right)\geq 0,\\ \lambda_{j},S_{i}^{k-},S_{i}^{e+},S_{i}^{u-}\geq 0, \end{array}\right. \end{array}$$

where $eff_{i}$, $x_{i}^{k}$ (k=1,2,3), $\lambda_{i}$, $y_{i}^{e}$ and $y_{i}^{u}$ represent the CEE, the *k*th input, the weighted coefficient, the desired output and the undesired output of Province *i* (DMU, i=1,2,…,30), respectively. $s_{i}^{k-}$, $s_{i}^{e+}$ and $s_{i}^{u-}$ denote the slack variables of the *k*th input, desired output and undesired output of Province *i*, respectively. If $eff_{i}<1$, it indicates that the CEE of Province *i* is low. Otherwise, the CEE has reached a relatively efficient state, and the greater the value, the higher the CEE of Province *i*.

#### 2.2.2. Construction of CEE Correlation Network

To better clarify the spatial linkage patterns of interprovincial CEE, this study applies a modified gravity model to measure the intensity of influence exerted by province *i* on province *j* with respect to CEE [46]:

(2) $$c_{ij}=\left\{\begin{array}{cc} \frac{G_{i}}{G_{i}+G_{j}}\times \frac{eff_{i}\times eff_{j}}{d_{ij}^{2}}, & i\neq j,\\ 0, & i=j, \end{array}\right.$$

where $c_{ij}$ denotes the CEE impact strength of Province *i* to Province *j*, reflecting the CEE impact pathway from *i* to *j*; $d_{ij}$ is the geographical distance between *i* and *j*; $eff_{i}$ and $G_{i}$ represent the CEE and GDP of Province *i*, respectively. Thus, the gravity matrix $C={\left(c_{ij}\right)}_{30\times 30}$ characterizing the spatial correlation effect of CEE among Chinese provinces is constructed. Using the 30 provincial administrative regions of China as nodes, a directed weighted CEE correlation network is established, where the directed edges represent interprovincial CEE correlations and the edge weights capture the corresponding influence intensities.

The resulting network is fully connected. In complex network theory, such networks often contain numerous weak connections or statistical noise, leading to false correlations and obscuring the identification of key interaction mechanisms. To mitigate redundancy and highlight essential structural connections, a threshold is typically applied to filter out insignificant edges. Taking the 2021 CEE correlation network as an example, this study sets the edge-weight threshold at 0.68, thereby eliminating approximately 80% of weak connections, which together account for about 10% of the total network weight. Consequently, the adjacency matrix $A={\left(a_{ij}\right)}_{30\times 30}$ of the undirected CEE correlation network is obtained, with its elements defined as:

(3) $$a_{ij}=a_{ji}=\left\{\begin{array}{cc} 1, & c_{ij}\;\;\mathrm{or}\;\;c_{ji}\geq 0.68,\\ 0, & \mathrm{otherwise}. \end{array}\right.$$

#### 2.2.3. Simplicial Complex Modeling of High-Order Interactions

Traditional complex network theory emphasizes pairwise connections but cannot capture the high-order interaction mechanisms of multi-agent collaboration. To address this limitation, this study adopts simplicial complex theory, extending the network structure to a higher dimensional level. In this framework, the fundamental interaction units are generalized into simplices, which allow the direct representation of non-pairwise relationships among nodes. As a special high-order structure, the simplicial complex has strict “inclusion” conditions: if a *d*-simplex (such as a 2-simplex formed by nodes i,j,k) exists, then all its subsets (i.e., (i,j)(i,k)(j,k) the three edges) must also exist as lower-order simplices in the network [47].

Based on this principle, the provincial carbon emission efficiency (CEE) correlation network in this paper is constructed through high-order modeling, with the basic network elements defined as follows:

- 0-simplex: A single node, representing an individual province.
- 1-simplex: An edge linking two nodes, signifying a significant CEE influence relationship between two provinces (pairwise interaction).
- 2-simplex: When each pair within a group of three provinces exhibits significant CEE influence relationships, they form a closed triangular structure, representing a tripartite coordination mechanism.

To quantitatively assess these high-order structures, the following topological metrics are introduced. The first-order adjacency matrix $A^{\left(1\right)}=A$ is used to encode pairwise (first-order) interactions. When there is a significant impact on the carbon emission efficiency of provinces *i* and *j*, the corresponding element $a_{ij}^{\left(1\right)}=1$; otherwise, it is 0. The first-order degree $k_{i}^{\left(1\right)}=\sum_{j=1}^{N}a_{ij}^{\left(1\right)}$ of node *i* (where *N* is the total number of nodes) represents the number of neighbors directly associated with province *i*. Correspondingly, the elements $a_{ij}^{\left(2\right)}=\sum_{k=1}^{N}b_{ijk}$ of the second-order adjacency matrix $A^{\left(2\right)}$ represent the number of second-order interactions that node *i* and *j* jointly participate in. $b_{ijk}$ reflects the high-order interaction of the triplet (second-order). When (i,j,k) forms a 2-simplex, $b_{ijk}=1$; otherwise, it is 0. The second-order degree of node $k_{i}^{\left(2\right)}=\frac{1}{2}\sum_{j,k=1}^{N}b_{ijk}$ represents the total number of 2-simplex in which province *i* is involved. In this study’s construction, 2-simplex are populated by closed triangles in the pairwise network. Consequently, their degree distribution demonstrates a pronounced “rich-get-richer” effect: nodes with larger first-order degrees tend to acquire disproportionately higher second-order degrees. This dynamic amplifies the degree heterogeneity of high-order interactions. Hence, the simplicial complex model offers a more powerful mathematical framework for capturing and analyzing the cross-regional linkages of CEE.

### 2.3. Analysis of Network Synchronization Stability

#### Multiorder Laplacian

Within the framework of high-order interactions, the synchronization stability of the network is governed not only by pairwise connections but also by multi-body cooperative structures. To assess the resilience of the CEE correlation network under external perturbations, a dynamical model based on multiorder Laplacians is developed. The first-order and second-order Laplacians, $L^{\left(1\right)}$ and $L^{\left(2\right)}$, respectively capture the effects of 1-simplex and 2-simplex on the system evolution [48]:

(4) $$L^{\left(1\right)}=D^{\left(1\right)}-A^{\left(1\right)},$$

(5) $$L^{\left(2\right)}=2D^{\left(2\right)}-A^{\left(2\right)},$$

where $A^{\left(1\right)}$ and $A^{\left(2\right)}$ represent the first-order and second-order adjacency matrices, respectively, and $D^{\left(1\right)}$ and $D^{\left(2\right)}$ are diagonal matrices, with the diagonal elements being the first-order degree $k_{i}^{\left(1\right)}$ and second-order degree $k_{i}^{\left(2\right)}$ of each node, respectively.

Furthermore, a multiorder Laplacian $L^{\left(mul\right)}$ is constructed to describe the coupling effects of different orders of interactions [48]:

(6) $$L^{\left(mul\right)}=\frac{1-\alpha }{\langle k^{\left(1\right)}\rangle }L^{\left(1\right)}+\frac{\alpha }{\langle k^{\left(2\right)}\rangle }L^{\left(2\right)},$$

where $\langle k^{\left(1\right)}\rangle$ and $\langle k^{\left(2\right)}\rangle$ represent the first-order and second-order average degrees, respectively; α∈[0,1] is the coupling parameter that controls the relative strength of the first-order and second-order effects. Particularly, α=0 and 1 respectively represent the first-order and second-order effects. Normalization processing ensures that the contributions of different orders can be comparable. The eigenvalues of $L^{\left(mul\right)}$ are sorted as follows: $0=\lambda_{1}\leq \lambda_{2}\leq \ldots \leq \lambda_{N-1}\leq \lambda_{N}$. The smallest non-zero eigenvalue $\mu =\lambda_{2}$ is used to measure the synchronization stability of the system: μ>0 indicates stable synchrony, and a larger value corresponds to a stronger resistance to perturbations.

### 2.4. Cross-Order Degree Correlation and Structural Optimization Strategies

The cross-order degree correlation is used to describe the statistical dependence between the first-order degree and the second-order degree [49]:

(7) $$DC=\mathrm{corr}\left(\left\{k_{i}^{\left(1\right)}\right\},\left\{k_{i}^{\left(2\right)}\right\}\right),$$

where $\left\{k_{i}^{\left(1\right)}\right\}$ and $\left\{k_{i}^{\left(2\right)}\right\}$ and denote the first-order and second-order degree sequences of all nodes, respectively. A high DC value implies that provinces with many direct connections also disproportionately control high-order interactions, potentially causing network centralization and reducing overall stability. To mitigate this, a directed second-order degree swap strategy is proposed to optimize the network structure:

**STEP 1:** Arrange nodes in descending order of their first-order degrees: $k_{1}^{\left(1\right)}>k_{2}^{\left(1\right)}>\ldots >k_{i}^{\left(1\right)}>\ldots >k_{N-1}^{\left(1\right)}>k_{N}^{\left(1\right)}$;
**STEP 2:** Pair the first and last nodes in the sorted sequence to form node pairs: (1,N),(2,N−1),…;
**STEP 3:** Sequentially swap the second-order degree of each node pair and calculate the values of μ(t) and DC(t) after the *t*-th swap.
**STEP 4:** Compute the increment of the smallest nonzero eigenvalue after the *t*-th swap: $\Delta \mu \left(t\right)=\mu \left(t\right)-\mu \left(t-1\right),\;t=1,\;2,\ldots ,\;\left[\frac{N}{2}\right]$. This increment reflects the marginal improvement in synchronization stability achieved by each structural adjustment.
**STEP 5:** Determine the optimal number of swaps $t^{*}$ and the corresponding coupling strength $\alpha^{*}$, that maximize Δμ(t), representing critical structural thresholds for strengthening network synchronization stability.

This approach improves the network’s overall resilience by strategically coupling core provinces (with high first-order degrees) with peripheral provinces (with low first-order degrees), counteracting the centralization tendency of high-order synergies. It thus offers a structural intervention mechanism to enhance regional collaborative efficiency.

## 3. Results

### 3.1. Spatiotemporal Evolution of High-Order Topological Structures

**Figure 1:**
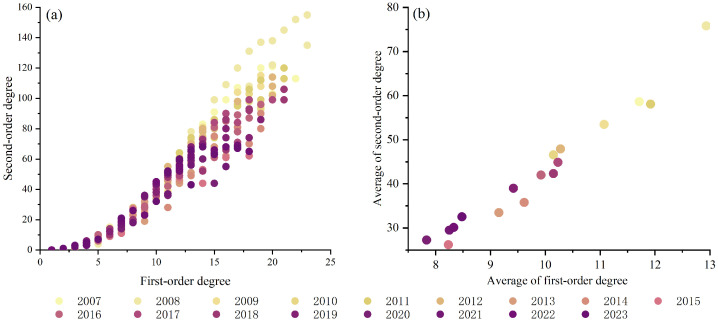
In the CEE correlation network from 2007 to 2023, (**a**) the relationship between second-order degree and first-order degree for each province, (**b**) the relationship between the average second-order degree and the averge first-order degree.

To uncover the high-order organizational mechanisms within the CEE correlation network among Chinese provinces, the first-order and second-order degrees of each provincial node were calculated, and their co-evolution patterns were analyzed. Figure 1a illustrates the relationship between second-order and first-order degrees for each province in China from 2007 to 2023, with colors transitioning from light to dark to represent successive years. The analysis reveals a strong positive correlation (Pearson correlation coefficient > 0.968), indicating that highly connected provinces dominate both pairwise interactions and high-order collaborations, thereby maintaining the continuity of the “core-periphery” structure in high-order interactions. Over time, the maximum values of both first-order and second-order degrees exhibit a declining trend, suggesting a gradual shift of the network from an initial single-pole aggregation dominated by a few core provinces toward a more balanced configuration with broader node participation. Moreover, Figure 1b depicts the annual average first-order and second-order degrees, which also show a continuous decrease over time, reflecting a reduction in the overall network connection density.

**Figure 2:**
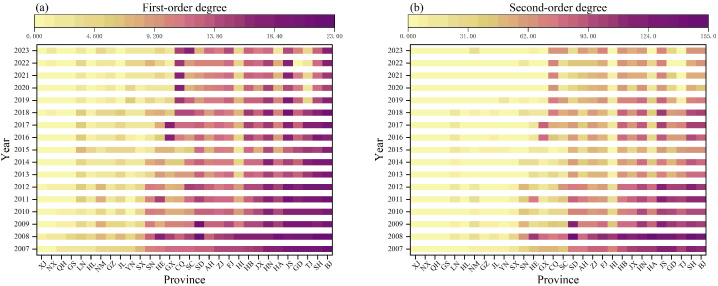
Heat maps of (**a**) first-order degrees and (**b**) second-order degrees in the CEE network among Chinese provinces, 2007–2023. Darker colors represent higher values.

To provide a more intuitive view of the evolution of spatial patterns, Figure 2 depicts the spatiotemporal distribution of heatmaps for first-order and second-order degrees from 2007 to 2023. Figure 2a shows that some economically developed regions in eastern and central China, such as Beijing, Shanghai, Jiangsu, Guangdong, and Tianjin, have consistently maintained high first-order degrees, forming a stable “network hub cluster”. In contrast, economically less developed regions in the northwest, including Xinjiang, Ningxia, Qinghai, and Gansu, have remained in the low-value range. Overall, this results in a clear gradient pattern of “high in the east, low in the west”. Notably, the extent of high-value areas has contracted over time, indicating that connection advantages are becoming increasingly concentrated in core economic regions.

Figure 2b illustrates that the spatial pattern of the second-order degree mirrors this differentiation. The number of provinces with high second-order degrees has decreased, and their values have diminished, while some central and western provinces, such as Chongqing, Guangxi, and Sichuan, have seen gradual increases. This evolution suggests that the initially highly concentrated tripartite collaborative structure is expanding into a broader yet moderately intense region, with the overall network structure gradually moving toward equilibrium.

### 3.2. The Synchronization Stability of the Network

Building on the multiorder Laplacian framework, this study assesses the synchronization stability of the CEE correlation network under external perturbations and investigates optimization strategies via cross-order degree correlations and a directed second-order degree swap approach. As shown in Equation (7), the degree correlation (DC) indicates a strong positive relationship between the first-order and second-order degrees in the original network (high DC value). This implies that provinces central in pairwise connections are also heavily involved in triadic synergistic structures. Such cross-order positive correlation may lead to reduced system stability, as the network becomes vulnerable to instability due to localized perturbations when a few core nodes simultaneously control multiple interaction modes.

**Figure 3:**
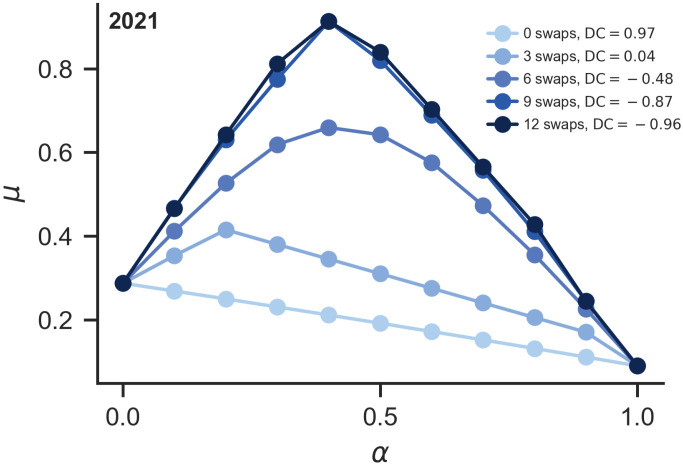
Cross-order degree correlation (DC) and synchronization stability (μ) under different numbers of swaps in the 2021 CEE correlation network.

To mitigate this, a directed second-order degree swap strategy is introduced, aimed at redistributing the participation opportunities of provinces in tripartite collaborative structures (2-simplex). Specifically, the strategy reduces the second-order degrees of provinces with high first-order degrees while increasing those of provinces with low first-order degrees. This approach disrupts the centralization of high-order interactions, thereby enhancing the overall synchronization stability of the network. Using 2021 as an example, Figure 3 illustrates the variation in the smallest nonzero eigenvalue across six swap scenarios, alongside the corresponding DC values. The results demonstrate that as more node pairs are swapped, the smallest nonzero eigenvalue steadily increases, indicating that increasing swaps promotes network decentralization and improves synchronization stability in systems combining pairwise and higher-order interactions.

**Figure 4:**
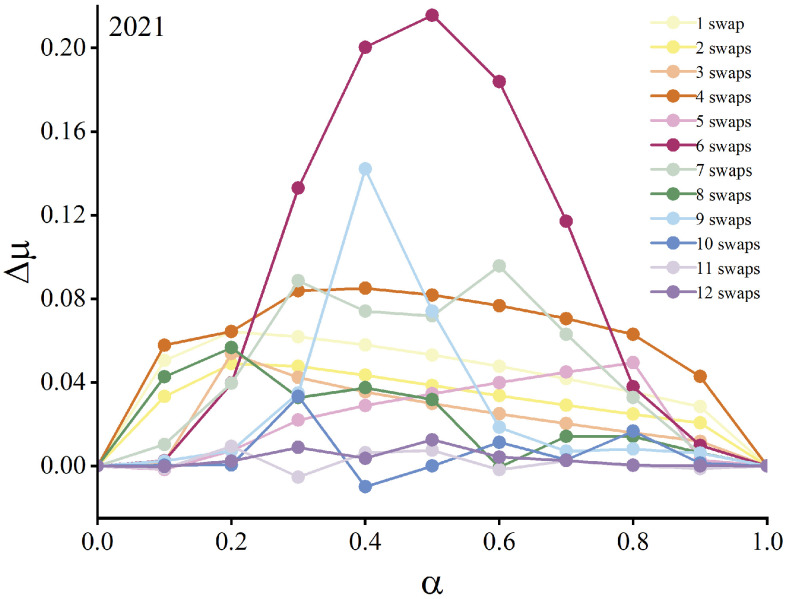
The relationship between Δμ and α and across varying numbers of swaps in the 2021 CEE correlation network.

Moreover, assessing the fluctuation range of the increment Δμ of the smallest nonzero eigenvalue between successive swaps provides a means to quantify the enhancement of network synchronization stability. Using the provincial CEE correlation network in 2021 as an example, Figure 4 shows the functional relationship between Δμ and α under different numbers of swaps. The results indicate that when six node-pair swaps are performed and the coupling parameter α=0.5, Δμ reaches its peak, representing the optimal improvement in synchronization stability. At this point, the contributions of first-order and second-order interactions are balanced, and the system attains a dynamic equilibrium between pairwise connections and triadic synergies. The six critical node-pair swaps identified are Jiangsu–Inner Mongolia, Chongqing–Shanxi, Beijing–Guangxi, Hubei–Liaoning, Hunan–Guizhou, and Shanghai–Yunnan. These pairings highlight a structural reorganization linking high-connectivity provinces with low-connectivity provinces, emphasizing the importance of fostering such cross-regional collaboration mechanisms to enhance the overall network’s resilience to perturbations.

### 3.3. The Optimal Network Structure

**Figure 5:**
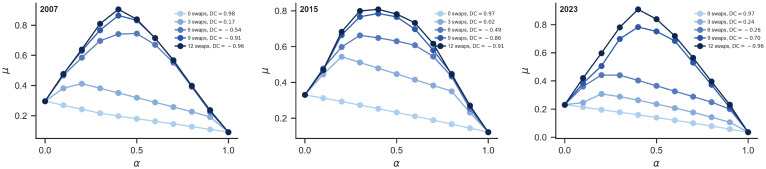
In the CEE correlation network, the cross-order degree correlation (DC) and synchronization stability (μ) under different numbers of swaps for the years 2007, 2015, and 2023.

**Figure 6:**
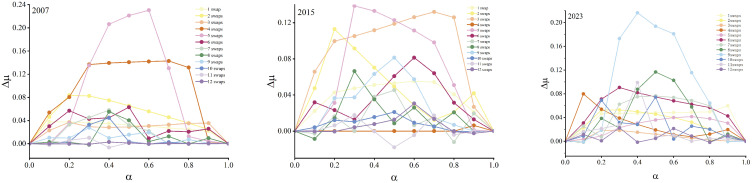
The functional relationship between Δμ and α in the CEE correlation network under varying numbers of swaps for the years 2007, 2015, and 2023.

**Figure A1:**
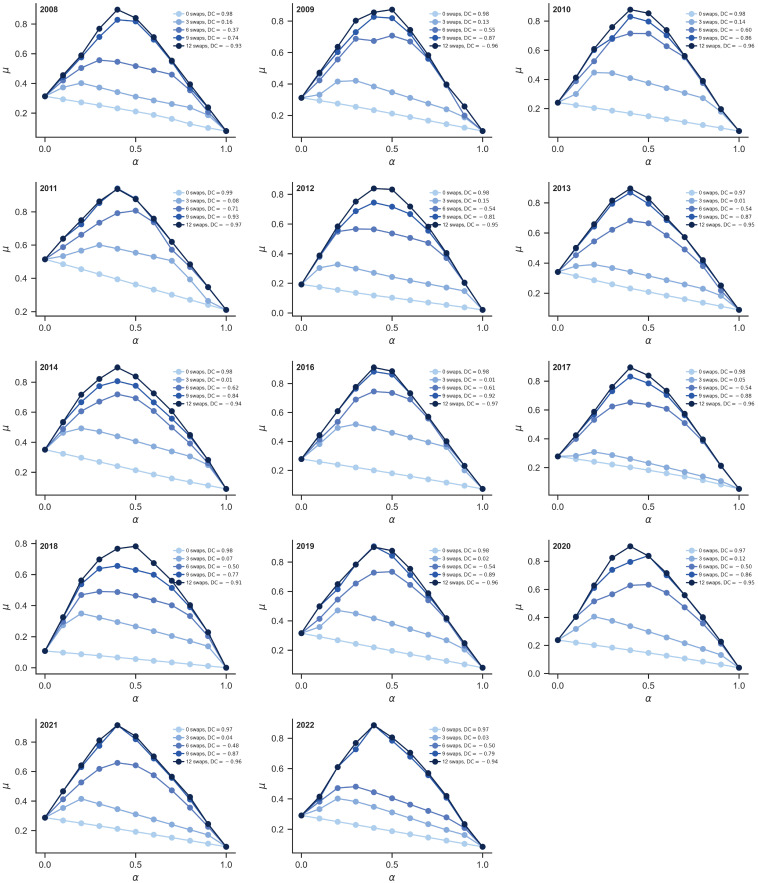
The cross-order degree correlation (DC) and synchronization stability (μ) under different numbers of swaps for the remaining years (from 2007 to 2023 excluding 2007, 2015, and 2023) in the CEE correlation network.

**Figure A2:**
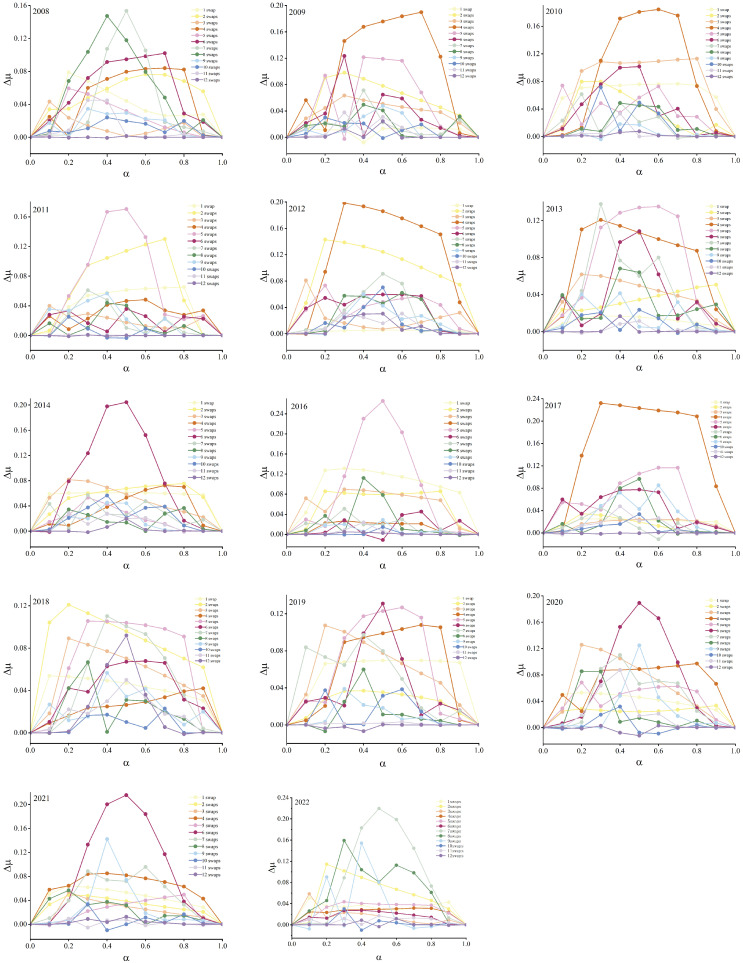
The functional relationship between Δμ and α under varying numbers of swaps for the remaining years (from 2007 to 2023 excluding 2007, 2015, and 2023) in the CEE correlation network.

To validate the broad effectiveness of the optimization strategy on network synchronization stability, this study systematically examines its evolution from 2007 to 2023. Figure 5 and Figure 6 present the results for the years 2007, 2015, and 2023, while the results for the remaining years are provided in Figure A1 and Figure A2 of Appendix A. A consistent trend is observed across all years: with an increasing number of swapped node pairs, the smallest nonzero eigenvalue (μ) steadily rises while the cross-order degree correlation (DC) value simultaneously declines, demonstrating the cross-period robustness of this optimization mechanism.

Based on the peak positions of Δμ in Figure 6, the optimal network structure parameters ($t^{*}$, $\alpha^{*}$) and the corresponding node pairs for each year were identified, as summarized in Table 1. The results indicate that the optimal number of swaps $t^{*}$ remains relatively stable, ranging from 4 to 7, with an average of approximately 5. The optimal coupling strength $\alpha^{*}$ fluctuates around 0.5, averaging roughly 0.45. These findings reveal a structure–function balance mechanism: in China’s provincial CEE correlation network, only a limited number of key structural adjustments are required to substantially enhance network stability. When the interaction strengths of first-order and second-order connections are comparable, the system achieves optimal synchronization. By performing second-order degree swaps among key provincial node pairs a limited number of times (around five), the cross-order degree correlation can be effectively reduced, and the smallest nonzero eigenvalue can be increased, thereby significantly improving network stability. Moreover, when the coupling strength ratio between first- and second-order interactions approaches 1:1 ($\alpha^{*}=0.5$), the network synchronization stability reaches the optimal level. This mechanism depends on the strategic redistribution of high-order collaborative resources between core provinces (high first-order degree) and peripheral provinces (low first-order degree), rather than merely increasing the number of connections.

**Figure 7:**
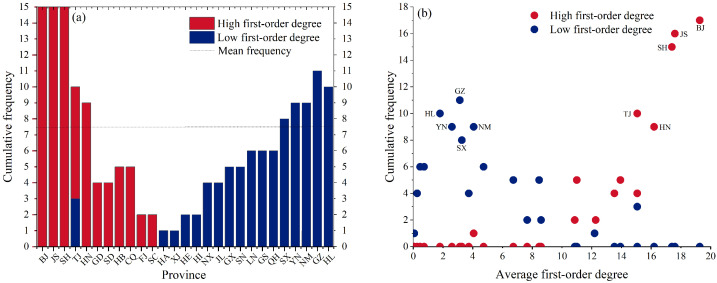
(**a**) Cumulative frequency of each province within optimal swapped node pairs during the observation period; (**b**) the relationship between cumulative frequency and average first-order degree.

Moreover, a statistical analysis of the optimal swapped node pairs presented in Table 1, illustrated in Figure 7a, reveals notable differences in the cumulative frequencies of participation in optimal swaps across provinces. Provinces with high first-order degrees such as Beijing, Jiangsu, Shanghai, and Tianjin exhibit substantially higher swap participation than the average, underscoring their enduring role as “high-order collaborative hubs”. In contrast, provinces with low first-order degrees, including Heilongjiang, Guizhou, and Inner Mongolia, frequently engage in swaps, highlighting their function as “collaborative potential nodes” in the structural optimization. Figure 7b further quantifies the relationship between each province’s cumulative swap frequency and its network topological attributes among the optimal swapped node pairs. Over the observation period, high first-order degree provinces show a significantly positive correlation between cumulative swap frequency and average annual first-order degree, whereas low first-order degree provinces exhibit a negative correlation. This demonstrates that the optimization strategy follows a clear selection principle, prioritizing the coupling of “high connectivity” with “low collaborative participation”, thereby mitigating the centralization tendency of the high-order network structure.

**Figure 8:**
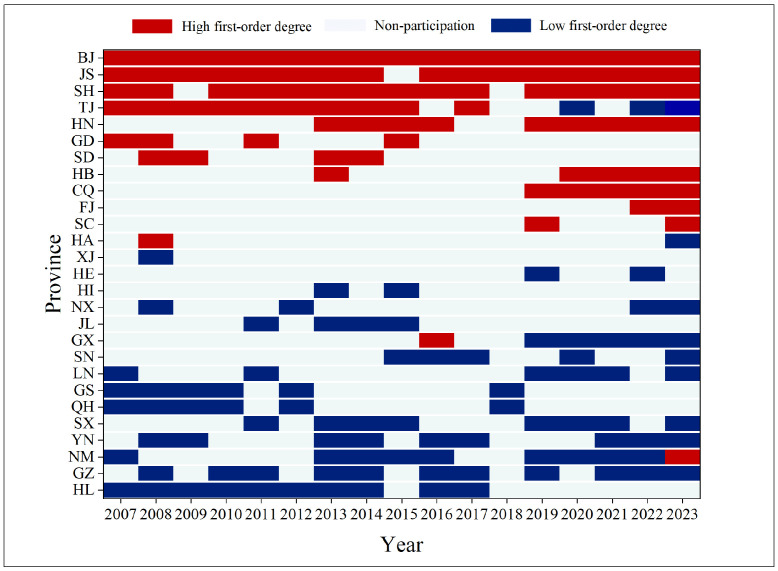
Spatial and temporal distribution of the optimal swapped node group during the observation period.

From the evolution of the optimal swapped node pairs (Figure 8), the high first-order degree group exhibits a pronounced core-periphery dynamic, forming a stable structure centered on Beijing, Jiangsu, and Shanghai. This highlights their strong connectivity and influential CEE radiation capabilities. Recently, Hubei and Chongqing have emerged as high-frequency swapped nodes, suggesting an increased role of the central region in cross-regional collaboration. In contrast, the low first-order degree group is primarily located in the northeastern, northwestern, and southwestern regions, displaying a dispersed spatial pattern. Notably, provinces with low first-order degrees that participate in swaps tend to rotate over time for instance, Qinghai and Gansu showed concentrated activity from 2007 to 2010, while Jilin did so from 2013 to 2015, indicating that network optimization must dynamically account for regional developmental differences.

## 4. Conclusions and Policy Implications

### 4.1. Main Conclusion

By integrating simplicial complex theory with the multiorder Laplacian dynamics model, this study achieves the modeling and optimization of the indecomposable tripartite collaborative mechanism within China’s CEE correlation network.

Empirical analysis reveals that the evolution of the CEE correlation network exhibits a transition from single-pole aggregation toward structural balance. A significant positive correlation between first-order and second-order degrees indicates that highly connected provinces dominate both pairwise and high-order interactions. Spatially, eastern developed provinces such as Beijing, Shanghai, Jiangsu, and Guangdong consistently serve as high-connectivity hubs. Temporally, while the maximum and average values of first-order and second-order degrees decline over time, the second-order degrees of central and western provinces, including Chongqing, Guangxi, and Sichuan, increase markedly. This shift suggests that higher-order interactions are extending beyond core provinces and driving the network toward a more decentralized and structurally balanced configuration.

Dynamic analysis using the multiorder Laplacian shows that the original network exhibits a high DC value, reflecting the over-concentration of higher-order interactions among core provinces that undermines system robustness. To address this, a directed second-order degree swap strategy is proposed to reallocate triadic interaction opportunities between high-order and low-order nodes. As the number of swaps increases, the minimum nonzero eigenvalue μ rises and DC declines, confirming that structural decentralization enhances network synchronization stability. The optimal swap frequency stabilizes between 4 and 7, and the optimal coupling strength $\alpha^{*}$ centers around 0.5. This indicates that network synchronization peaks when first-order and second-order interactions are equally weighted. Notably, this equilibrium arises from structural recoupling between core and peripheral provinces, demonstrating that targeted, limited interventions can substantially improve system resilience.

Statistical analysis shows that high first-order degree provinces Beijing, Jiangsu, Shanghai, and Tianjin frequently appear in optimal swaps, reinforcing their role as high-order synergistic hubs. Conversely, low-degrees provinces such as Heilongjiang, Guizhou, and Inner Mongolia are often selected as nodes with latent synergistic potential. Since 2019, Hubei and Chongqing have shown increased participation, reflecting the growing influence of central regions in cross-regional carbon collaboration.

### 4.2. Policy Implications

Based on the findings, this study proposes the following policy recommendations to enhance the stability of the regional CEE collaborative system through targeted structural interventions.

First, establishing collaborative alliances between core and peripheral provinces can promote high-order cooperation mechanisms. Drawing on the identified optimal swapped nodes, targeted carbon collaboration alliances, such as Jiangsu–Inner Mongolia or Shanghai–Yunnan, should be developed, focusing on green technology transfer, carbon sink development, and energy interconnection. This approach can effectively align the influence of highly connected provinces with the ecological and developmental potential of peripheral regions.

Second, formulating specialized policies to reallocate high-order synergies can reduce the system’s dependence on core nodes. A national-level cross-regional collaborative innovation fund is recommended to prioritize support for provinces with low first-order degrees, enabling their participation in triadic or higher-order collaborative projects that enhance carbon emission efficiency. This strategy can increase their involvement in 2-simplex, reduce the DC value, and strengthen the network’s resilience to disturbances.

Third, implementing differentiated carbon governance strategies enhances the functional adaptation of nodes. Provinces with high first-order and second-order degrees, such as Beijing, Jiangsu, and Shanghai, should reinforce their leadership roles in regional carbon policy coordination, standard setting, and technology dissemination. Conversely, provinces with lower connectivity require increased fiscal transfers, ecological compensation, and capacity-building initiatives to improve their integration into high-order networks and prevent long-term structural marginalization.

Fourth, promoting region-specific functional roles and synergistic interactions can achieve spatial structural balance. Southeastern coastal provinces like Jiangsu and Shanghai should leverage their advantages in carbon markets and the digital economy to extend green influence to central and western regions. Central provinces such as Hubei and Chongqing should accelerate the creation of regional green technology hubs to absorb and redistribute CEE influence. Northwestern and northeastern provinces, including Qinghai and Heilongjiang, can integrate into multilateral cooperation through ecological compensation mechanisms, carbon sink trading systems, and renewable energy production, transforming ecological value into collaborative benefits.

The strategy proposed in this study not only applies to systems with specific properties but can also be extended to any complex system characterized by interactions among elements. Nevertheless, several limitations remain. First, the goals for CEE improvement ultimately require implementation across various sectors during production, necessitating the further decomposition of provincial-level targets to the sector level. Compared with provincial-level analysis, identifying the role of higher-order synergies in shaping the stability of the CEE correlation network at the sector level will facilitate more precise implementation of CEE improvement policies. Second, how to further quantify the enhanced stability of the interprovincial CEE correlation system into specific economic or environmental outcomes remains a key issue that urgently needs to be addressed in future research. Third, due to the availability of official statistical data, the provincial data used in this study are currently updated to 2023. As official data are progressively released, subsequent research can further validate and extend the findings presented here.

## Figures and Tables

**Table 1:** Optimal network structural parameters and swapped node pairs (2007–2023).

Year	($t^{*}$, $\alpha^{*}$)	Optimal Swapped Node Pairs
2007	(5, 0.6)	(BJ,QH), (TJ,LN), (SH,HL), (GD,GS), (JS,NM)
2008	(7, 0.5)	(BJ,XJ), (SD,QH), (SH,NX), (HA,HL), (TJ,GZ), (GD,YN), (JS,GS)
2009	(4, 0.7)	(SD,GS), (BJ,QH), (TJ,YN), (JS,HL)
2010	(4, 0.6)	(BJ,HL), (TJ,GZ), (SH,GS), (JS,QH)
2011	(5, 0.5)	(BJ,GZ), (JS,HL), (TJ,JL), (SH,SX), (GD,LN)
2012	(4, 0.3)	(BJ,GS), (TJ,QH), (SH,NX), (JS,HL)
2013	(7, 0.3)	(BJ,HL), (SH,YN), (TJ,SX), (HN,NM), (JS,GZ), (SD,JL), (HB,HI)
2014	(6, 0.5)	(BJ,YN), (SH,SX), (TJ,NM), (HN,HL), (JS,GZ), (SD,JL)
2015	(5, 0.3)	(BJ,SX), (TJ,HI), (SH,NM), (HN,SN), (GD,JL)
2016	(5, 0.5)	(SH,HL), (BJ,YN), (JS,GZ), (GX,SN), (HN,NM)
2017	(4, 0.3)	(BJ,GZ), (SH,HL), (JS,YN), (TJ,SN)
2018	(2, 0.2)	(BJ,GS), (JS,QH)
2019	(6, 0.5)	(JS,SX), (BJ,NM), (HN,HE), (CQ,LN), (SC,GZ), (SH,GX)
2020	(6, 0.5)	(CQ,SX), (BJ,NM), (HN,LN), (JS,GX), (HB,SN), (SH,TJ)
2021	(6, 0.5)	(JS,NM), (CQ,SX), (BJ,GX), (HB,LN), (HN,GZ), (SH,YN)
2022	(7, 0.5)	(BJ,GX), (JS,NX), (CQ,TJ), (HN,NM), (HB,GZ), (SH,HE), (FJ,YN)
2023	(9, 0.4)	(BJ,GZ), (SC,LN), (CQ,HA), (JS,NX), (FJ,SN), (NM,SX), (SH,GX), (HB,YN), (HN,TJ)

Note: $t^{*}$ denotes the number of swaps that maximizes Δμ(t), $\alpha^{*}$ represents the corresponding coupling strength, and in the node pairs, the first entry indicates the province with high first-order degree, while the second entry denotes the province with low first-order degree.

## Data Availability

All the data used in this study are derived from the following publicly available authoritative statistical materials and databases: Data on fixed-asset investment and GDP were collected from the China Statistical Yearbook, while labor force and energy consumption figures were obtained from the China Labor Statistical Yearbook and the China Energy Statistical Yearbook, respectively. Carbon emissions data were sourced from the CEADs database (https://www.ceads.net/, accessed on 22 February 2026). Inter-provincial distances were calculated using the R language in combination with the geographical coordinates of provincial capitals.

## Appendix A

**Table A1:** Provincial administrative regions (PARs) and abbreviations.

PAR	Abbr.	PAR	Abbr.
Anhui	AH	Jiangxi	JX
Beijing	BJ	Jilin	JL
Chongqing	CQ	Liaoning	LN
Fujian	FJ	Ningxia	NX
Gansu	GS	Inner Mongolia	NM
Guangdong	GD	Qinghai	QH
Guangxi	GX	Shaanxi	SN
Guizhou	GZ	Shandong	SD
Hainan	HI	Shanghai	SH
Hebei	HE	Shanxi	SX
Heilongjiang	HL	Sichuan	SC
Henan	HA	Tianjin	TJ
Hubei	HB	Xinjiang	XJ
Hunan	HN	Yunnan	YN
Jiangsu	JS	Zhejiang	ZJ

## References

[1] Wang Y., Guo C., Chen X., Jia L.Q., Guo X.N., Chen R.S., Zhang M.S., Chen Z.Y., Wang H.D. (2021). Carbon peak and carbon neutrality in China: Goals, implementation path and prospects. China Geol..

[2] Jiang B., Raza M.Y. (2023). Research on China’s renewable energy policies under the dual carbon goals: A political discourse analysis. Energy Strategy Rev..

[3] Luo G., Guo J., Yang F., Wang C. (2023). Environmental regulation, green innovation and high-quality development of enterprise: Evidence from China. J. Clean. Prod..

[4] IEA (2025). CO2 Emissions. Global Energy Review 2025.

[5] Xue L., Zheng Z., Meng S., Li M., Li H., Chen J.M. (2022). Carbon emission efficiency and spatio-temporal dynamic evolution of the cities in Beijing-Tianjin-Hebei Region, China. Environ. Dev. Sustain..

[6] Liu C., Sun W., Li P., Zhang L., Li M. (2023). Differential characteristics of carbon emission efficiency and coordinated emission reduction pathways under different stages of economic development: Evidence from the Yangtze River Delta, China. J. Environ. Manag..

[7] Zhao X., Long L., Yin S., Zhou Y. (2023). How technological innovation influences carbon emission efficiency for sustainable development? Evidence from China. Resour. Environ. Sustain..

[8] Choi Y., Tang Z. (2025). Urbanization and the Bipolarization of Carbon Emission Efficiency Across Chinese Cities. Sustainability.

[9] Lan J., Wang P. (2025). An efficiency perspective on low carbon pilot city policy and carbon emission performance of listed enterprises: Quasi-experimental evidence from China. Energy Econ..

[10] Benini G., Enstad E., Mersha A.A., Rossini L. (2026). Technical versus environmental efficiency in steel production: A global perspective. J. Environ. Manag..

[11] Addis A.K. (2025). Sustainability and efficiency analysis of 42 countries: Super SBM-DEA model and the GML productivity index with undesirable outputs. Ecol. Indic..

[12] Chen M., Wang Q., Bian X., Zhao Y. (2025). Research on the impact of low-carbon pilot policies on the measurement of carbon emission efficiency of industrial enterprises. Process Saf. Environ. Prot..

[13] Yan Y., Ma D., Hu C., Zhang F., Deng P., Li K. (2026). Analyzing the carbon emission efficiency and influencing factors of China’s thermal power generation sector based on super-SBM and ESTDA models. Carbon Balance Manag..

[14] Chen A., Duan H., Li K., Shi H., Liang D. (2025). A Three-Stage Super-Efficient SBM-DEA Analysis on Spatial Differentiation of Land Use Carbon Emission and Regional Efficiency in Shanxi Province, China. Sustainability.

[15] Singh A., Mishra S. (2025). Operational efficiency and service quality of Indian electricity distribution utilities: A three-stage DEA and Malmquist Index analysis. Util. Policy.

[16] Luo R., Wang N. (2026). Carbon emission quota allocation for 280 Chinese cities: Integrating machine learning and DEA with regional heterogeneity. Expert Syst. Appl..

[17] Liu X., Sun F., Li Y. (2026). The impact of new quality productive forces on urban carbon emission performance in the Yangtze river economic belt of China. Sci. Rep..

[18] Cheng Z., Nie X., Zhong X. (2025). How climate policy uncertainty affects carbon emission efficiency: Evidence from Chinese Prefecture-level cities. J. Asia Pac. Econ..

[19] Debbarma J., Kumar V., Ekundayo D. (2025). Measuring carbon emission efficiency in a developing country: A comparative study of sustainability initiatives and nonsustainability initiatives of manufacturing firms. Bus. Strategy Environ..

[20] Amowine N., Li H., Baležentis T., Štreimikienė D. (2025). Technology innovation and carbon efficiency in Africa: What is the role of digitalization and digital inclusive finance?. Technol. Econ. Dev. Econ..

[21] Ding R., Liang J. (2026). Research on Synergistic Co-Promotion Mechanism and Influencing Factors of Science and Technology Finance Efficiency and Carbon Emission Efficiency from the Perspective of Multi-Layer Efficiency Networks. Systems.

[22] Jiang H., Lu J., Zhang R., Liu Y., Li P., Xiao X. (2026). Promoting or Inhibiting? The Nonlinear Impact of Urban–Rural Integration on Carbon Emission Efficiency: Evidence from 283 Chinese Cities. Land.

[23] Chen X., Wang R., Szalmane Csete M., Sun Y., Hu S. (2025). Assessment of carbon emission efficiency in China’s construction industry based on an innovative “efficiency-space-time” integrated model. Eng. Constr. Archit. Manag..

[24] Yao H., Yu X., Mao H., Zhang H., Thompson R. (2025). Logistics hub policies and carbon emission efficiency: Insights into emission reduction in China. Environ. Dev. Sustain..

[25] Siyiti M., Yao X. (2024). Natural resource assets management and urban carbon emission efficiency: Evidence from quasi-natural experiment in China. Energy Econ..

[26] Estrada E., Hatano N., Benzi M. (2012). The physics of communicability in complex networks. Phys. Rep..

[27] Chasman D., Siahpirani A.F., Roy S. (2016). Network-based approaches for analysis of complex biological systems. Curr. Opin. Biotechnol..

[28] Hu X., Dong G., Christensen K., Sun H., Fan J., Tian Z., Gao J., Havlin S., Lambiotte R., Meng X. (2025). Unveiling the importance of nonshortest paths in quantum networks. Sci. Adv..

[29] Pujol J.M., Flache A., Delgado J., Sangüesa R. (2005). How can social networks ever become complex? Modelling the emergence of complex networks from local social exchanges. J. Artif. Soc. Soc. Simul..

[30] Wei X., Chen B. (2024). Spatial association network structure of agricultural carbon emission efficiency in Chinese cities and its driving factors. Sci. Rep..

[31] Zhang L., Wang H., Guo B., Liu X., Deng C., Zhao Z., Jiang X., Li Y. (2025). Characteristics and formation mechanism of carbon emission efficiency spatial correlation network: Perspective from Shandong Province. Ecol. Indic..

[32] Cheng H., Wu B., Jiang X. (2024). Study on the spatial network structure of energy carbon emission efficiency and its driving factors in Chinese cities. Appl. Energy.

[33] Du R., Zhang N., Zhang M., Kong Z., Jia Q., Dong G., Tian L., Ahsan M. (2024). Identifying the optimal node group of carbon emission efficiency correlation network in China based on pinning control theory. Appl. Energy.

[34] Petri G., Expert P., Turkheimer F., Carhart-Harris R., Nutt D., Hellyer P.J., Vaccarino F. (2014). Homological scaffolds of brain functional networks. J. R. Soc. Interface.

[35] Patania A., Petri G., Vaccarino F. (2017). The shape of collaborations. EPJ Data Sci..

[36] Grilli J., Barabás G., Michalska-Smith M.J., Allesina S. (2017). Higher-order interactions stabilize dynamics in competitive network models. Nature.

[37] Xie H., Ding B. (2026). Topological persistence pinpoints higher-order network vulnerabilities. Chaos Interdiscip. J. Nonlinear Sci..

[38] Lucas M., Gallo L., Ghavasieh A., Battiston F., De Domenico M. (2026). Reducibility of higher-order networks from dynamics. Nat. Commun..

[39] Sun H., Radicchi F., Bianconi G. (2026). Triadic percolation on multilayer networks. Phys. Rev. E.

[40] Battiston F., Bick C., Lucas M., Millán A.P., Skardal P.S., Zhang Y. (2026). Collective dynamics on higher-order networks. Nat. Rev. Phys..

[41] Gao Z., Ghosh D., Harrington H.A., Restrepo J.G., Taylor D. (2023). Dynamics on networks with higher-order interactions. Chaos Interdiscip. J. Nonlinear Sci..

[42] Liu H., Shen D., Dabić M., Lu J. (2025). A novel methodology for risk assessment considering risk higher order interactions and propagation effects. IEEE Trans. Eng. Manag..

[43] Bick C., Gross E., Harrington H.A., Schaub M.T. (2023). What are higher-order networks?. SIAM Rev..

[44] Wang Q., Zhou P., Zhou D. (2012). Efficiency measurement with carbon dioxide emissions: The case of China. Appl. Energy.

[45] Zhang J., Zeng W., Wang J., Yang F., Jiang H. (2017). Regional low-carbon economy efficiency in China: Analysis based on the Super-SBM model with *CO*_2_ emissions. J. Clean. Prod..

[46] Zhang R., Tai H., Cheng K., Zhu Y., Hou J. (2022). Carbon emission efficiency network formation mechanism and spatial correlation complexity analysis: Taking the Yangtze River Economic Belt as an example. Sci. Total Environ..

[47] Muolo R., Giambagli L., Nakao H., Fanelli D., Carletti T. (2024). Turing patterns on discrete topologies: From networks to higher-order structures. Proc. A R. Soc..

[48] Lucas M., Cencetti G., Battiston F. (2020). Multiorder Laplacian for synchronization in higher-order networks. Phys. Rev. Res..

[49] Zhang Y., Lucas M., Battiston F. (2023). Higher-order interactions shape collective dynamics differently in hypergraphs and simplicial complexes. Nat. Commun..

